# Influence of Service Conditions and Mix Design on the Physical–Mechanical Properties of Roller-Compacted Concrete for Pavement

**DOI:** 10.3390/ma17030552

**Published:** 2024-01-23

**Authors:** Julián Pulecio-Díaz, Miguel Sol-Sánchez, Fernando Moreno-Navarro

**Affiliations:** 1 Faculty of Engineering, Universidad Cooperativa de Colombia, Ibague 730006, Colombia; 2 Laboratory of Construction Engineering, University of Granada, 18071 Granada, Spain; msol@ugr.es (M.S.-S.); fmoreno@ugr.es (F.M.-N.)

**Keywords:** physical–mechanical properties, relative humidity, roller-compacted concrete, temperature

## Abstract

This research focuses on the behavior of roller-compacted concrete (RCC) used in pavements, which are prone to deterioration affecting their performance. These deteriorations result from various causes, including traffic load, errors during construction, mix design, and ambient conditions. Among these, ambient conditions could lead to a marked variable impact on material behavior and durability depending on the conditions associated with each region. Accordingly, this study aims to deepen the understanding of the effect, which a broader range of ambient conditions and different mix designs have on the physical and mechanical properties of RCC. Measurements such as the amount of water vapor per kilogram of air were used to apply the findings comprehensively. The RCC analysis encompassed experimentation with different compositions, altering the cement water ratio amount, and adding a superplasticizer. The impact of curing on the materials was evaluated before subjecting them to various humidity and temperature conditions. Laboratory tests were conducted to measure performance, including moisture, shrinkage, compressive strength, and the progression of flexural fracture resistance over curing periods of up to 90 days. The results revealed a logarithmic correlation between shrinkage and ambient humidity, which is the most determining factor in performance. Mix optimization through increased cement and reduced water enhanced the tensile strength of the material. Furthermore, the curing process was confirmed to increase resistance to shrinkage, especially in the long term, establishing it as a crucial element for the structural stability of RCC, which is relatively insensitive to variations in ambient conditions.

## 1. Introduction

Recent research in pavements has achieved notable progress, especially regarding flexible and rigid pavements, including the development of structural design methods ranging from empirical to mechanistic approaches; contributions from north American and European methodologies are emphasized. Moreover, innovative and alternative materials focused on sustainability and durability have been introduced [1]. These materials offer benefits, such as pollution reduction, self-repair capabilities, and support for implementing and loading autonomous and electric vehicles. They have also been developed to facilitate user interaction and efficient information transmission for road infrastructure managers, aiming to optimize road maintenance and conservation. This includes developments from functional to structural aspects. Notably, the application of surface stiffness tests [2] used to evaluate the existing load capacity of pavements and the design of new pavements stands out. There is also the option to conduct an exhaustive analysis of existing pavements using traditional back-calculation methods, which rely on measuring the pavement surface deflection under known loads. Then, when advanced multi-level back-calculation techniques are used, even greater accuracy is achieved, incorporating optimization algorithms to determine the parameters of the different pavement layers more precisely [3]. Furthermore, innovative methods, which reduce carbon emissions and energy consumption regarding construction techniques, have been implemented.

Nonetheless, in the last decade, the study of the materials and structural design of roller-compacted concrete for pavement (RCC) [4,5,6] has substantially increased due to the current needs of the industry and commerce sectors, leading to a higher volume of vehicle mobility [7,8]. Roller-compacted concrete pavement (RCCP) has broadly been used in (i) industrial plants and parking access roads; (ii) intermodal shipping yards, ports, and loading docks; (iii) truck/freight terminals [9,10], bulk commodity storage, and distribution centers; (iv) low-volume urban [11,12] and rural roads; (v) aircraft parking areas [13]; (vi) long- or short-term military loading zones, forward or rearward bases of operation, and airfields; (vii) recreational vehicle pad storage; (viii) vehicle maintenance and compost areas; (ix) large commercial parking lots; (x) public park roadways; (xi) timber and logging operation roads; (xii) highway shoulders; and (xiii) temporary travel lanes of rapid construction to divert traffic. Hence, due to the vehicular loads channeled in extreme climates [14,15,16,17] and the low or no vehicle travel speeds [18], a flexible pavement, due to its weakness, would be inadequate for the service life of these scenarios.

RCC mixes for pavement have the advantage of containing less cement quantity [19] than the standard structural concrete used in other civil engineering applications. Thus, they generate less carbon footprint and become a more sustainable solution [20] compared to conventional concrete. However, their applicability is not yet at the level of flexible pavements, since they still require studies related to key factors, such as the need for improving the texture and roughness of RCCP related to skid resistance and noise, among others [21,22], and their resistance to cracking and climate affectations in the short and long terms [23,24,25,26]. State-of-the-art methods have shown the behavior of RCCP under different curing conditions, relating temperature to compressive strength and shrinkage [4,27,28,29,30]. In particular, it is known that shrinkage due to higher ambient temperature weakens the RCCP, leading to a less durable material with higher susceptibility to failure due to climate actions [31,32].

Conversely, other studies have stated that this phenomenon depends on factors such as the design and composition of the mixes used. For example, Gholami and Modarres [33] found that using a certain superplasticizer could modify the resistance of concrete to shrinkage. At the same time, the quantity of water during RCC manufacturing was also found to have a substantial impact on material behavior during the shrinkage phenomenon [34]. Moreover, field studies determined that shrinkage stresses depend on other climate actions, causing variable material performance (mainly in the initial construction hours) due to the difficulty in maintaining stable ambient conditions [23]. However, most of these previous studies focused on assessing only a few specific ambient conditions, and they still need to correlate the impact of these and the effect of modifying fundamental design parameters, such as cement content. In addition, previous research has mainly focused on studying the behavior of the material after 90 days of curing by submersion [35] to delete the effects of the concrete not maturating and its suction capacity through initial pores [36]. These aspects contribute to the knowledge gap in RCC behavior during the initial curing hours when the most variable and influential phenomenon occurs.

In this context, the study analyzes the impact of several service conditions (relative humidity, temperature, and grams of water vapor per kilogram of air) and various mix designs (different cement, water, and additive contents) on the RCC performance, focusing on the shrinkage phenomenon and resistance to cracking. These parameters were selected due to their relevance in establishing the durability of the material [37,38,39]. In addition, the impact on compressive strength and modulus of rupture as quality control were assessed. Accordingly, this research aims to study the physical–mechanical variables with a more significant influence on the behavior of RCC for pavement, seeking to provide the knowledge for selecting the most appropriate design and construction conditions mix, depending on the application area with different ambient conditions [40]. Simultaneously, this study aims to generate essential data for calibrating laboratory models, which accurately replicate the real-world conditions of pavement materials. This task is vital for an effective pavement analysis and design, as it allows considering the impacts of moisture and shrinkage through the finite element method analysis [32,35]. This approach contrasts with the usual focus on more conventional parameters, such as material stiffness, thickness, adherence conditions between layers, and vehicle load modeling. While these aspects are important, they often overshadow the significant influence of moisture and shrinkage on pavement behavior and durability.

## 2. Materials and Methods

### 2.1. Materials

Three types of RCC mixes were studied to analyze the effect of selected ambient conditions depending on the cement quantity used to manufacture these and the influence of adding a superplasticizer. The mixes assessed were (1) standard RCC [41] with 12% cement, without admixture (referred to in this article as RCC 12C); (2) standard RCC with 16% cement (denoted as RCC 16C); and (3) standard RCC with 12% cement, with addition of a high-activity superplasticizer per water-reducing admixture (named as RCC 12C admixture). The cement dosage was selected in the range commonly used in commercial RCC [41]. The superplasticizer dose for the third mix was 0.5% of the cement weight, as indicated by the manufacturer (Master Builders Solutions, Beachwood, OH, USA).

Cement type CEM II/A-M [42], classified as a composite, forms the basis of the material used. It integrates a mixture, which varies in the range of 80–88% clinker and 12–20% granulated blast furnace slag (GGBFS), together with additions such as silica fume, pozzolana, fly ash, calcined ash, and limestone, with a proportion of up to 5% of other minor components. The material establishes specific restrictions on its chemical composition, such as sulfate content, which must be 4.0% or less, expressed as SO_3_. It also limits the chloride content, which must not exceed 0.1%. In the physical aspect, it is characterized by a setting time of no less than 60 min, a volume stability of no more than 10 mm, and a specific gravity of 3.15. Mechanically, it stands out with a nominal compressive strength of 42.5 MPa.

**Figure 1:**
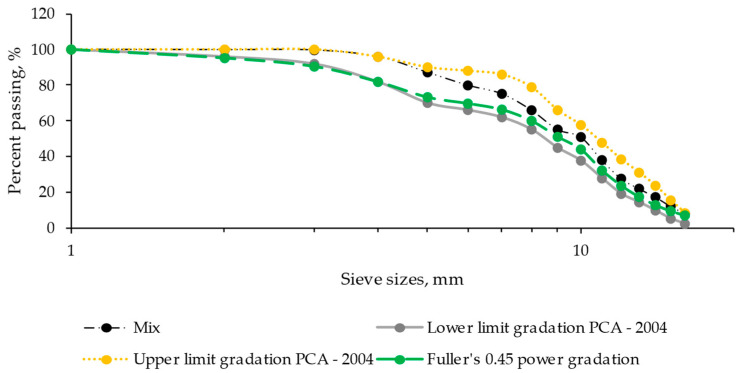
Gradation for roller-compacted concrete pavement, according to PCA (2004).

In terms of the aggregates, a calcareous type with a combined specific gravity of 2.775 was used. This value is further specified with specific gravities of 2.840, 2.805, 2.805, and 2.732 for aggregate fractions of 16/25, 10/16, 5.6/10, and 4/5.6, respectively [43,44]. The combined water absorption is recorded at 0.472%, with individual values of 0.460, 1.020, 0.946, and 0.153% for the mentioned aggregate fractions. The aggregates conform to the particle size spectrum defined by the Portland Cement Association in 2004 [45,46,47], as illustrated in Figure 1.

**Figure 2:**
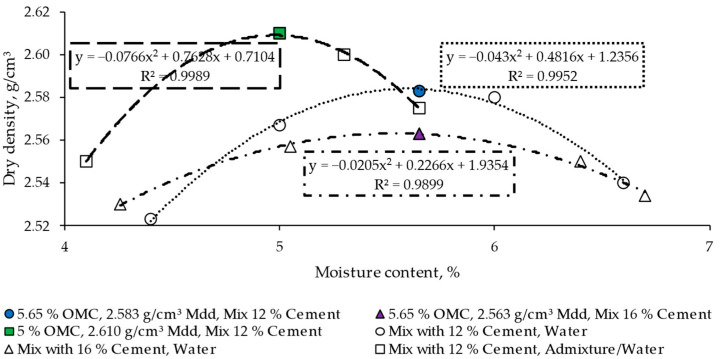
Moisture–density curves. OMC: optimum moisture content, Mdd: maximum dry density.

The optimum moisture content (OMC) to manufacture the mixtures without admixture was 5.65% [48], and the maximum dry density (Mdd) ranged from 2.563 to 2.583 g/cm^3^; these parameters were defined by the proctor compaction method (ASTM D1557−12) [49] (Figure 2), and they allow for the assessment of the influence of cement (between 12% and 16%) without varying the water content while evaluating the impact of reducing the water content from 5.65 to 5.00% by using a superplasticizer.

The mix proportions were established using the mix proportioning procedure based on the soil compaction analogy method. This method involves calculating the components of RCC in kilograms, cubic meters, and liters per cubic meter. The components calculated include the cement, the oven-dry weight of aggregates, the saturated surface-dry (SSD) weight of aggregates, absorbed water, water at OMC, free water at OMC, admixtures, and an air content of 1.5%, all correlated with the water–cement ratio. Details of the mixes used in the investigation are available for reference in Table 1, Table 2 and Table 3.

### 2.2. Testing Plan

**Figure 3:**
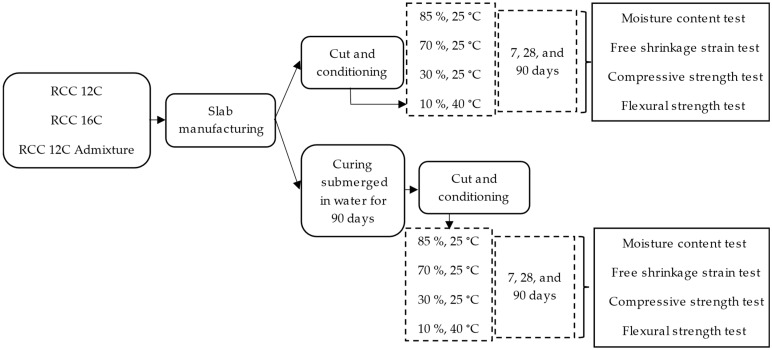
Testing plan.

In this research, the service conditions and mix design were analyzed considering the physical–mechanical properties of RCC pavements. The procedure followed consists of various steps graphed in Figure 3.

Following the testing plan, the laboratory work included manufacturing specimens in molds of 100 mm × 300 mm × 300 mm. This procedure consisted of (1) mold preparation, (2) RCC mix fabrication, and (3) RCC mix compaction into two equal parts with a compaction period of 1 min (total procedure time of 4 min). The compaction procedure was carried out with a plate compactor system NLT 173. This standard was used because it reflects a compaction process adequate for the project objectives [50,51]. Moreover, it represents the field compaction system for asphalt mixes and RCC. After the RCC mix hardened, the concrete slabs (specimens) were cut with a cutting system to provide cubic (100 mm × 100 mm × 100 mm) and beam-formed (100 mm × 100 mm × 285 mm) specimens for laboratory tests.

The specimens of each mixture type (RCC 12C, RCC 16, and RCC 12C Admixture) were separated into two groups: (1) those to be directly conditioned at different ambient conditions (specific RH and temperature) and (2) those to be subjected to a curating process by submersion in water at 23 ± 2 °C for 90 days before being conditioned at different ambient conditions (specific RH and temperature).

**Figure 4:**
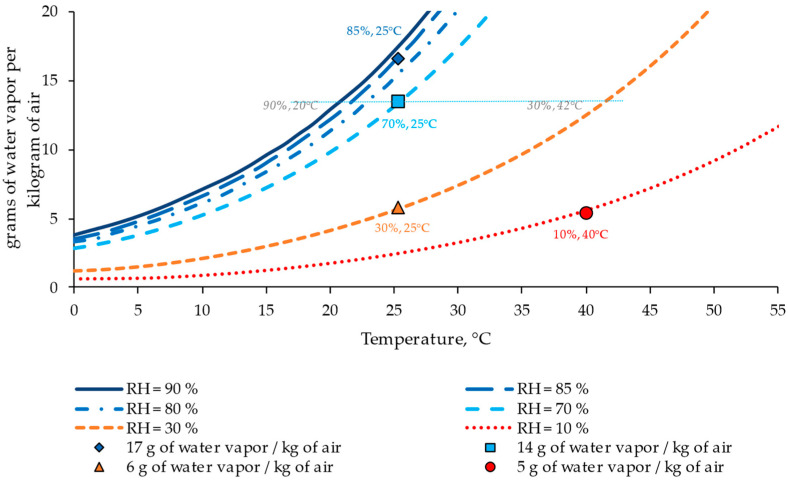
Grams of water vapor per kilogram of air according to diverse combinations of relative humidity (RH) and temperature (T). Source: Ref. [40].

Subsequently, four ambient conditions, i.e., four combinations of relative humidity at two temperatures, were selected: (i) 85%, 25 °C; (ii) 70%, 25 °C; (iii) 30%, 25 °C; and (vi) 10%, 40 °C. The conditioning periods were established on days 0, 7, 28, and 90. This conditioning allows for the influence of humidity at the same temperature to be assessed while analyzing the effect of temperature for similar water conditions. These conditions were selected to correlate the results with the grams of water vapor per kilogram of air in the atmosphere (Figure 4). These measurements represent multiple ambient conditions and allow for a more comprehensive application of the results. Figure 4 shows an example of how a condition with 70% relative humidity (RH) and a temperature of 25 °C corresponds to around 14 g of water vapor per kilogram of air but is equivalent to other conditions with approximately 90% RH and 20 °C, and 30% RH and 42 °C.

In the testing plan (Figure 3), the following tests were carried out for each sample: (i) moisture content employing a gravimetric test, (ii) shrinkage evaluation, (iii) compression strength, and (iv) flexion modulus using the three-point loading test to assess the resistance to cracking.

**Figure 5:**
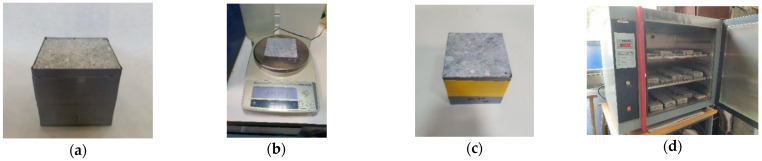
Cube samples used for the moisture content test: (**a**) samples with gray duct tape; (**b**) weighing of the 10 mm piece; (**c**) thin and thick pieces sealed together with yellow tape; (**d**) samples in the oven.

The moisture content of each specimen was determined with a gravimetric test [35,52] at intervals of 0, 7, 28, and 90 days in three replicates per ambient condition. The 100 mm × 100 mm × 100 mm cubes were used for this test. A thin piece of 10 mm × 100 mm × 100 mm was cut utilizing an adequate cutting system, and the 10 mm lateral surfaces were sealed using gray duct tape and weighted (initial weight). The other cube piece (90 mm × 100 mm × 100 mm) was also sealed with gray duct tape on all its lateral surfaces. Afterward, both pieces (the thin and thick pieces) were placed one on top of the other by their unsealed surfaces. Yellow tape was used to maintain this position. The specimens were subjected to different ambient conditions for a specific control period (7, 28, and 90 days). Then, the thin and thick pieces were separated to measure the relative moisture evolution in the thin piece. At the end of this process, all specimens were placed in an oven (INDELAB, Murcia, Spain) at 110 ± 5 °C to measure dry weight and determine the absolute moisture evolution of the specimens. This process is presented in Figure 5.

**Figure 6:**
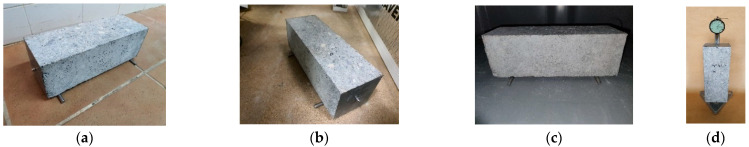
A beam sample conditioned at different ambient conditions (**a**–**c**). Free shrinkage strain test (**d**).

The beams cut from the slabs were used to evaluate the shrinkage susceptibility in the specimens. First, the lateral faces of the beam were sealed with duct tape (Figure 6a,b) to avoid tension effects on its surface and internal compression effects [35,37], which occur during the drying process. Therefore, shrinkage (ASTM C157/C157M-17) [53] was measured at days 0, 7, 28, and 90 using a fixed frame with a displacement meter (ELE International, Milton Keynes, UK), i.e., periodically measuring the free shrinkage strain of each specimen exposed to the ambient conditions established. Figure 6 shows a summary of the procedure.

**Figure 7:**
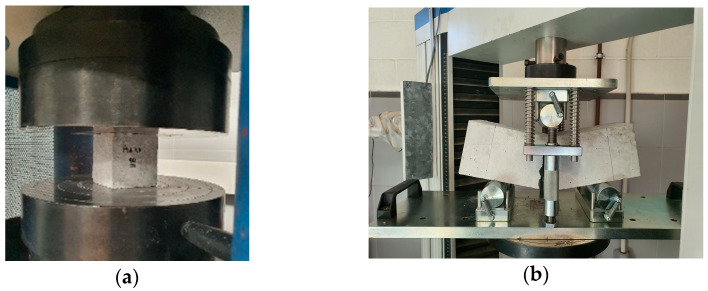
Strength tests of roller-compacted concrete: (**a**) compressive strength test; (**b**) flexural strength test.

The compressive strength (Ibertest, Madrid, Spain) for cubic specimens (ASTM C39/C39M-21 and BS EN 12390-3:2019-TC) [54,55] and the modulus of rupture (Ibertest, Spain) of the beams with the center-point loading test (ASTM C293/C293M-16) [56] were established to evaluate the structural performance of each RCC after diverse conditioning states (Figure 7). In the first test, the loading rate employed was 0.25 MPa/s, and the cubes selected were previously subjected to conditioning in various ambient conditions for 90 days. In the second case, the loading rate was 0.02 MPa/s, and the beams selected were previously subjected to conditioning in various ambient conditions for 90 days.

## 3. Results and Discussion

The analysis of the results and discussion are divided into different sections to evaluate (i) the effect of the ambient conditions (temperature and relative humidity), (ii) the impact of the mix design on the performance depending on the grams of water vapor per kilogram of air in the atmosphere, and (iii) the influence of the curing conditions through the tests performed, i.e., moisture content, shrinkage, compression strength, resistance to flexion failure–cracking. In addition, a parametric analysis is presented at the end of this section to provide the weight of each factor on the performance of the materials assessed.

### 3.1. Effect of Ambient Conditions

**Figure 8:**
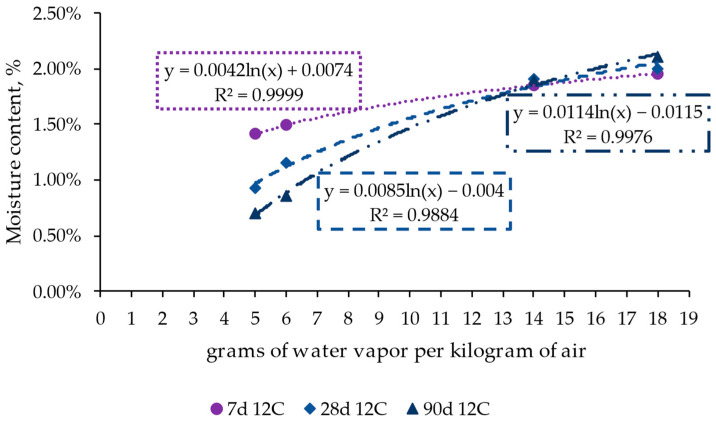
Grams of water vapor per kilogram of air versus moisture content on days 7, 28, and 90 in the roller-compacted concrete mix with 12% cement (reference mix).

Figure 8 presents the influence of ambient conditions on the evolution of the moisture content among the selected periods, i.e., 7, 28, and 90 days, for the case of RCC with 12% cement and without admixture. This graph displays the moisture content variation in relation to the change in ambient water content (expressed as grams of water vapor per kilogram of air), comparing the cases with 5, 6, 14, and 18 grams of water vapor per kilogram of air. Five grams represents the condition of 10% RH and 40 °C, six grams 30% RH and 25 °C, fourteen grams 70% RH and 25 °C, and eighteen grams represents 85% and 25 °C. These values allow for analyzing the influence of the temperature by comparing the cases with 5 and 6 g (similar relative humidity but different temperatures) and the effect of humidity by comparing the cases with 6, 14, and 18 g of water vapor per kg of air. Because this analysis uses this variable, it could allow for a broader interpretation of the results for other ambient conditions.

The results show a logarithmic relationship between the ambient condition and the change in moisture content of the specimens, indicating that this factor plays an essential role in the state of the concrete. In this sense, the results show a limited influence of ambient temperature compared to the cases in which the relative humidity changed, emphasizing the cases with 30% and 70% relative humidity.

On the other hand, the results show that the moisture content remained relatively constant in the first seven days when the curve tended to be flat. However, from that point on, the values decreased remarkably in the cases with higher temperature and lower humidity (represented by the values of lower grams of water vapor per kilogram of air). The results stated that the most significant differences were seen in the medium and long terms (beyond 28 days) for the cases between 30% and 70% humidity, despite being at the same conditioning temperature. This denotes that temperature could affect the performance of the concrete in the long term but with a lower impact than other ambient parameters, such as humidity.

**Figure 9:**
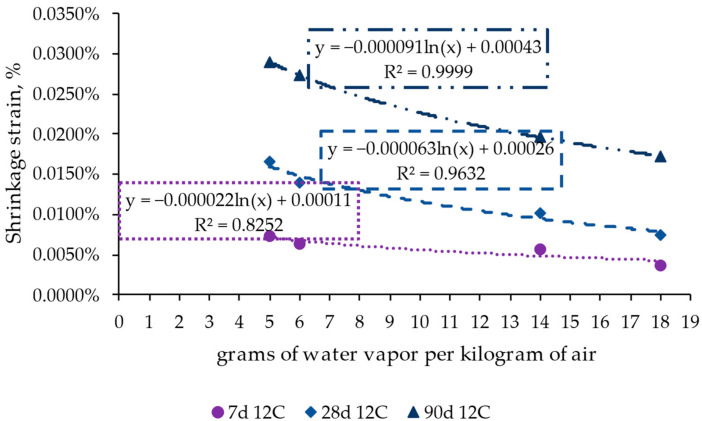
Influence of ambient conditions measured in grams of water vapor per kilogram of air on shrinkage strain in the roller-compacted concrete mixes with 12% cement (reference mix) for days 7, 28, and 90.

Figure 9 displays the correlation between the grams of water content per kilogram of air with changes in the shrinkage strain for different periods (7, 28, and 90 days, as representative times) to analyze the influence of ambient conditions on concrete performance. This analysis is shown for the case of the reference RCC with 12% cement. In consonance with the previous results, ambient conditions had little influence on the shrinkage values at times shorter than seven days. However, in the long term, the strain in the specimens under dry conditions was around double compared to those under wet conditions, highlighting the relevance of ambient conditions following an exponential/logarithmic trend.

Additionally, the results confirmed that temperature affects the behavior of the concrete, leading to an acceleration in strain when increasing the temperature from 25 °C to 40 °C under dry conditions, represented by the cases of 5 and 6 g of water vapor per kg of air, respectively. However, this fact was less accentuated than the case of passing from 30% of RH to 70%. These changes were more remarkable in the long term, yielding differences between wet and dry conditions for the same temperature and around 45–60% RH. Therefore, the most influential parameter for concrete performance was the water content of the atmosphere, which correlates with the shrinkage property. This allows for extrapolating the results for a wider range of temperature–humidity conditions, which could take place during the service life of roller-compacted concrete pavements.

**Figure 10:**
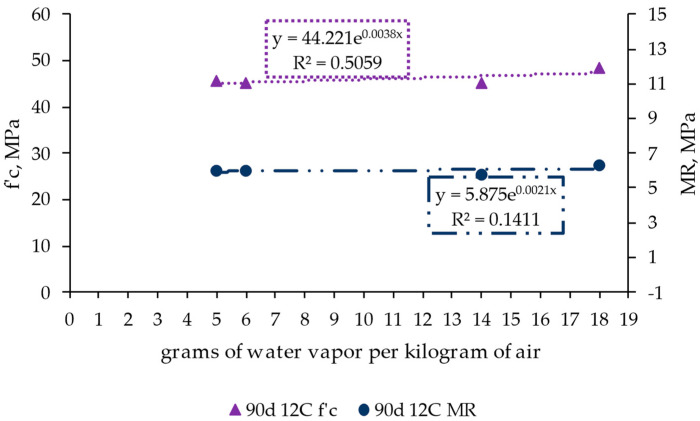
Influence of ambient conditions measured in grams of water vapor per kilogram of air on concrete strength for days 7, 28, and 90 in the roller-compacted concrete mix with 12% cement (reference mix).

Figure 10 shows the influence of the ambient temperature–humidity (through grams of water vapor per kilogram of air) on the structural performance of the RCC by representing the values of compression strength (f’c, in MPa) and the modulus of rupture at flexion stress (MR, in MPa) after 90 days of conditioning. This period was selected to assess these parameters in consonance with the previous results, showing that it corresponds to the most representative assessment time.

The results show that the studied ambient conditions had little influence on the material strength under compression and flexural efforts. Only a slight increase in material strength was obtained with higher relative humidity. This denotes that the ambient conditions strongly impact a phenomenon like shrinkage but have little influence on strength at the assessed periods.

### 3.2. Impact of the Mix Design on the Performance under Different Ambient Conditions

**Figure 11:**
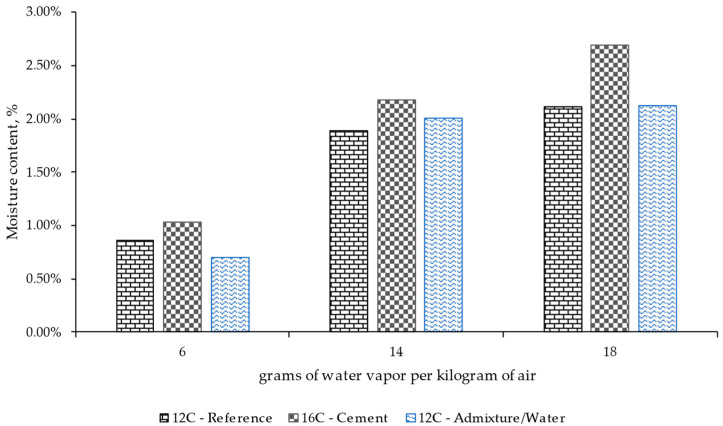
Influence of the cement mix design and the use or not of a superplasticizer on the moisture content of the specimens after 90 days of conditioning under different ambient conditions (grams of water vapor per kilogram of air).

Figure 11 presents the values recorded for each material with different moisture contents depending on the quantity of cement (12% versus 16% cement) and the use of an admixture/water (only with RCC with 12% cement) to analyze the influence of the mix design. This evaluation was carried out after 90 days under various ambient conditions (6, 14, and 18 g of water vapor per kg of air). These periods and ambient conditions were selected according to previous results. This graph also shows that the concrete mix with a higher cement dosage led to a higher moisture content value, regardless of the ambient condition. This indicates that the RCC 16C design allows for obtaining a material with a higher capacity to retain moisture over time, which could, in turn, allow for better hardening and maturation of the concrete. This fact was generally more accentuated under wet conditions, where higher ambient humidity was reproduced.

**Figure 12:**
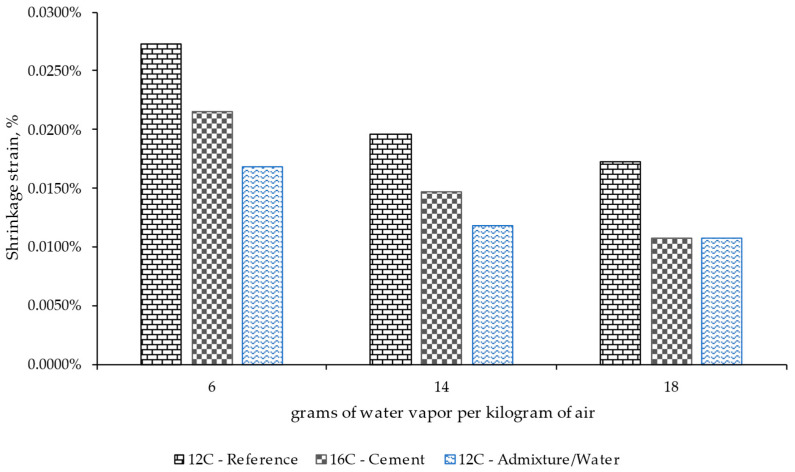
Effect of the cement mix design and the use or not of a superplasticizer on the shrinkage strain measured for the specimens after 90 days of conditioning under different ambient conditions (grams of water vapor per kilogram of air).

Figure 12 presents the mean values of shrinkage strain measured for the specimens with different concrete mixes after 90 days of conditioning under various ambient conditions. The results confirm that the shrinkage phenomenon decreased when the ambient humidity for all the materials assessed increased. This was more remarkable for the mixes with higher cement dosage and the superplasticizer (admixture), since those materials provided a lower trend in shrinkage strain. For the first case, this could be related to the higher capacity of this material to retain water, as seen previously, compared to the case with lower cement content. However, both used the same quantity of water during manufacturing. Regarding the case of the mix with the admixture, the results indicated how this solution could improve the resistance of RCC to shrinkage, which could be related to the use of lower water dosage during the manufacturing of the material.

**Figure 13:**
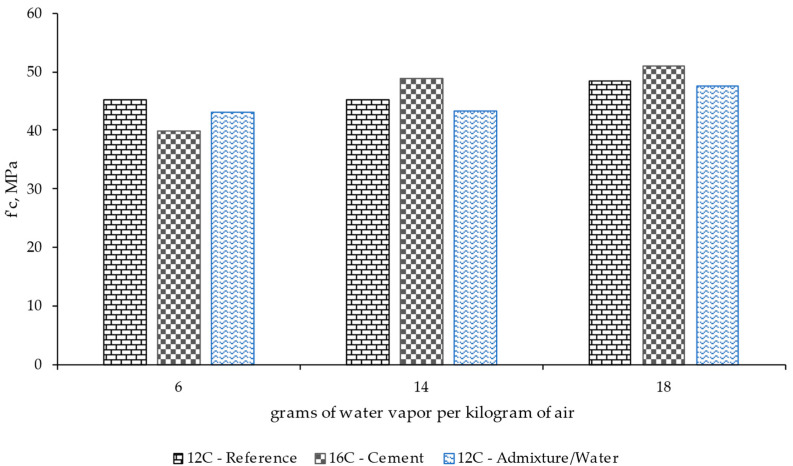
Compression test results for different mix designs and the use or not of a superplasticizer after 90 days of conditioning under different ambient conditions (grams of water vapor per kilogram of air).

**Figure 14:**
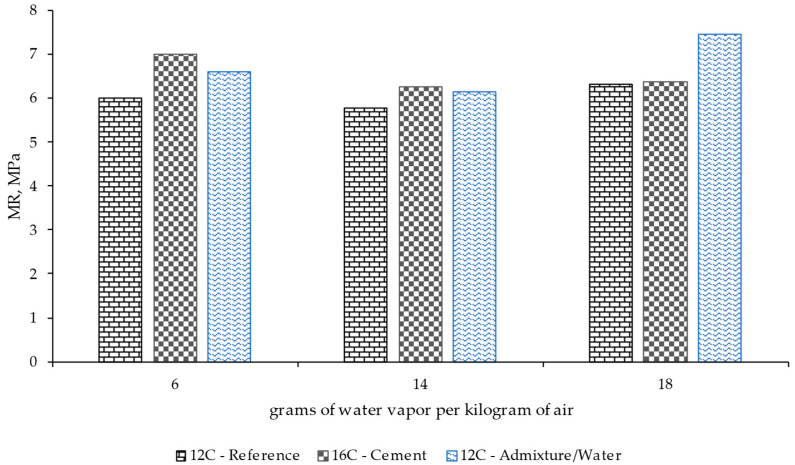
Modulus of rupture results for different mix designs and the use or not of a superplasticizer after 90 days of conditioning under different ambient conditions (grams of water vapor per kilogram of air).

Figure 13 and Figure 14 show the mean compression and flexural strength results recorded for each mixture under various conditioning conditions for 90 days. Generally speaking, the material with higher cement dosage showed a slight increase in resistance, but such difference was limited compared to the analysis of other properties, such as shrinkage.

### 3.3. Impact of Concrete Curing

**Figure 15:**
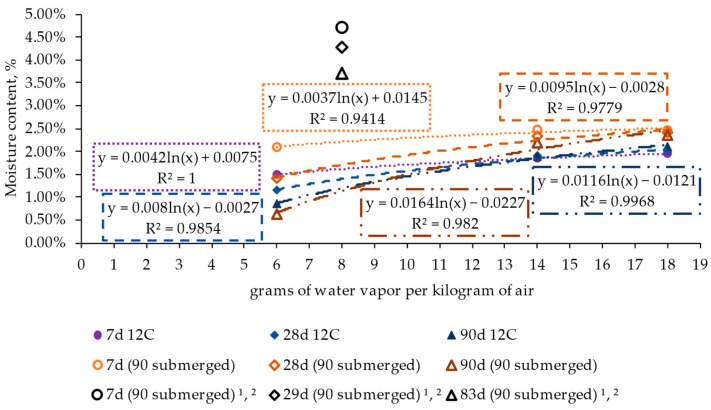
Influence of the curing process on the moisture content evolution of specimens. ^1^ [35], ^2^ [37].

**Figure 16:**
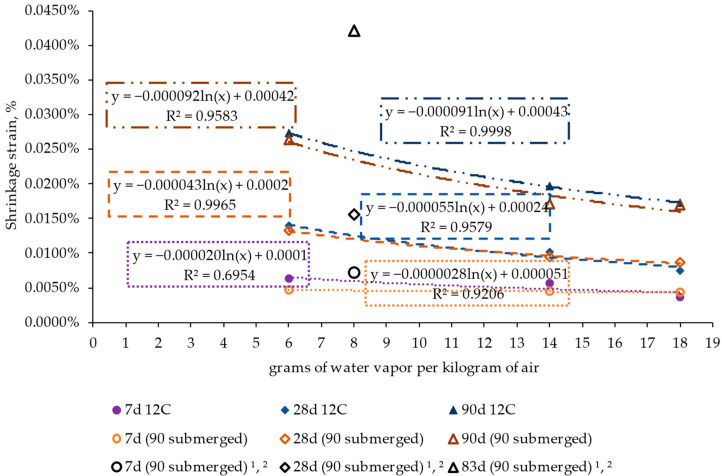
Impact of curing the material on the results of shrinkage of the specimens at different conditioning periods and under various ambient conditions. ^1^ [35], ^2^ [37].

**Figure 17:**
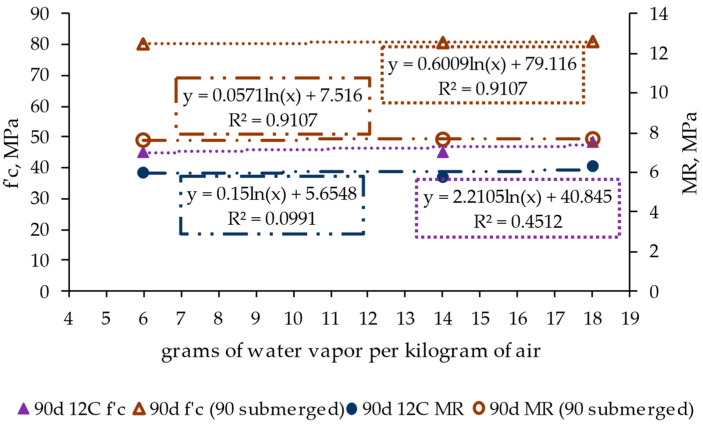
Effect of the curing process on the structural strength of the roller-compacted concrete at different conditioning periods and under various ambient conditions. f’c: compressive strength, MR: modulus of rupture.

To assess the influence of applying a curing process to the material, consisting of submerging the specimens in water at 23 ± 2 °C for 90 days prior to being conditioned to different ambient conditions, Figure 15, Figure 16 and Figure 17 present the mean values recorded for the reference material (12% cement without admixture) with and without being subjected to the curing process. This analysis was carried out for different conditioning processes expressed in grams of water vapor per kilogram of air, corresponding to each ambient condition.

The results of moisture content evolution (Figure 15) show that the curing process had a short-term influence (in the first seven days), leading to higher values of water content in the concrete for all ambient conditions. In addition, this trend persisted over time for the wet state cases (represented as the cases around 18 g of water vapor per kg of air), where reduced variation was observed over the 90-day period. However, in the dry condition cases, the evolution over time strongly impacted both specimens with and without being subjected to the curing process, leading to quite similar results for both situations after 90 days.

The current findings are consistent with previous research (Figure 15), such as that conducted by Jafarifar et al. [35,37]. In those studies, the alteration in moisture content in an atmosphere with 40% relative humidity and a temperature of 25 °C, corresponding to 8 g of water vapor per kg of air, led to a variation of 9–27% over a period ranging from 7 to 83 days.

Figure 16 shows the shrinkage values measured for the specimens subjected and not subjected to the curing process. The results indicate that the curing process had little influence on the shrinkage process, regardless of the period, i.e., after 7, 28, and 90 conditioning days, and the ambient humidity. These results contrast with those related to structural strength (compression and flexural strength) shown in Figure 17, where the curing process clearly led to higher strength values (nearly 80% for the compression results and around 35% for the flexion results). Therefore, this indicates that the curing process had a notable influence on the structural strength of the material and the evolution of moisture content but a limited effect on the trend of the material toward shrinkage.

The results align with the findings of previous studies, such as those by Jafarifar et al. [35,37]. These studies found that in an atmosphere with 40% relative humidity and a temperature of 25 °C, corresponding to 8 g of water vapor per kg of air, the variation in drying shrinkage ranged from 54 to 487% over a period ranging from 7 to 83 days (Figure 16).

### 3.4. Parametric Analyses of Service Conditions and Mix Design

**Figure 18:**
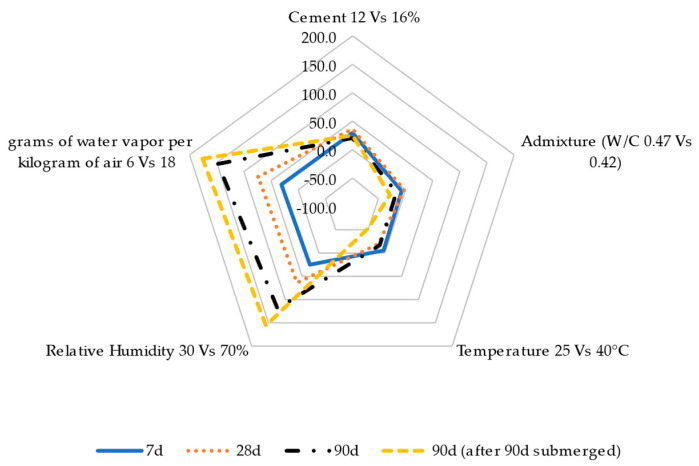
Parametric analyses of the moisture content considering diverse factors.

**Figure 19:**
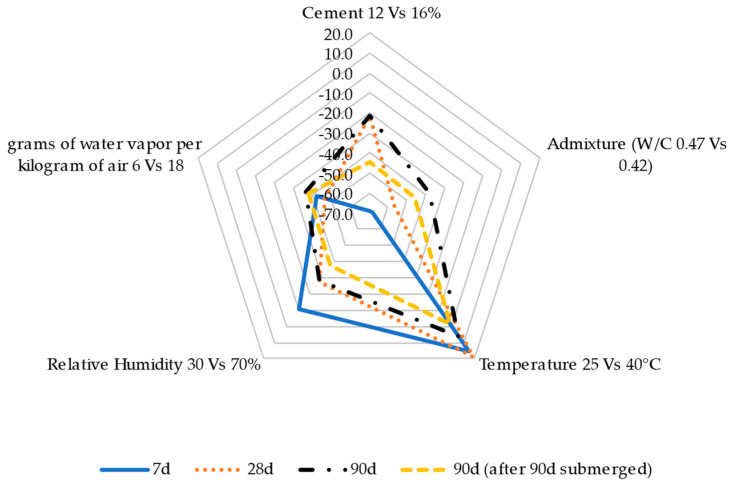
Parametric analyses evaluating the influence of the mix design and ambient conditions on roller-compacted concrete shrinkage.

**Figure 20:**
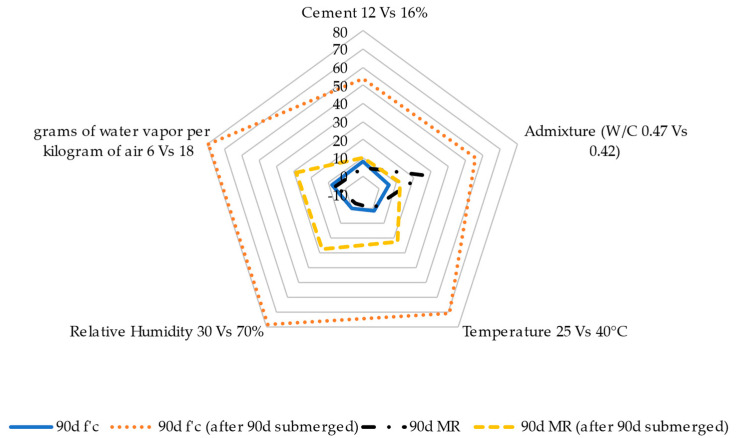
Parametric analyses of compressive strength (f’c) and modulus of rupture (MR) versus design and ambient parameters. W/C: water–cement ratio.

Based on the previous results, Figure 18, Figure 19 and Figure 20 show a parametric analysis to determine the most influential variables in the evolution of the properties of the RCC mixes, such as moisture content (Figure 18), shrinkage strain (Figure 19), and compression/flexion strength (Figure 20). The results are expressed as a percentage of change when comparing the mix with 12 and 16% cement content, the use of a superplasticizer in comparison with the reference RCC without admixture, the increase in ambient temperature from 25 to 40 °C, the increase in relative humidity from 30 to 70%, and the change in ambient conditions from 6 to 18 g of water vapor per kg of air.

The results in Figure 18 indicate that the mix design and conditioning temperature had limited influence on the variation in moisture content in the specimens. In contrast, the ambient humidity had the highest impact on this property. This was more notable when the conditioning period was increased and the curing process was applied.

This agrees with the shrinkage results shown in Figure 19, where the ambient humidity had a notable impact on reducing the concrete strain trend in a wet atmosphere, particularly in the long term and when subjecting the material to a curing process. In this case, in contrast to the previous results, the mixture design influenced this property (comparable effect to the presence of wet conditions), leading to a reduction in shrinkage when using a higher cement dosage while reducing the water dosage during manufacture using a superplasticizer. Generally speaking, these trends were slightly accentuated when the curing process was applied.

Regarding the results of the structural performance of the RCC, Figure 20 shows that the design and ambient parameters had little influence on the resistance properties, highlighting the notable impact of the curing process.

## 4. Conclusions

The current research assessed the influence of ambient service conditions and mix design factors on the performance of RCC, evaluating properties such as evolution in moisture content, shrinkage strain, compression strength, and resistance to flexion failure. This was assessed for different conditioning periods, studying the influence of ambient factors, such as temperature and relative humidity, on materials with varying cement and water quantities (using a superplasticizer) in the manufacturing process. Based on the results, the following conclusions were drawn:

- A wet atmosphere with humidity values higher than 70% gave the specimen a higher capacity to retain moisture over the studied period, resulting in shrinkage values around half of those recorded under dry conditions, with relative humidity lower than 30%.
- The shrinkage strain showed a logarithmic relationship with the variation in grams of water vapor per kilogram of air, allowing for the prediction of RCC behavior under a wide combination of humidity–temperature values represented through such parameters. Conversely, this factor had little influence on other RCC properties, such as compression strength and resistance to flexion failure, where the ambient conditions had a limited impact on material strength.
- The design of RCC with a higher dosage of cement led to a higher capacity to retain moisture content in specimens, leading to a reduction in the shrinkage trend. Nonetheless, this reduction in material strain was more remarkable when manufacturing the RCC using a superplasticizer, which allowed for a reduction in water dosage. In all mix designs, little influence on structural strength was observed despite the variations in material design.
- The curing process had a higher impact on specimens under dry conditions, leading to a higher capacity to retain water and improve the performance of the material. Curing specimens under wet conditions had a limited effect but allowed for preserving the moisture retained by the material.
- The influence of the curing process was more accentuated in the structural properties, allowing for an increase in the properties of the material while contributing slightly to a reduction in the shrinkage strain in RCC in the long term.
- The most relevant factors for the shrinkage property were the use of RCC under wet conditions, improving the design of the RCC using higher cement dosage, and above all, using a superplasticizer, which allowed reducing the water content during manufacturing and thus reducing the shrinkage trend.
- The possibility of expanding this research to analyze how the physical–mechanical properties of the RCC mix influence the service conditions is recommended, considering the incorporation of other admixtures in everyday applications. In addition, the inclusion of sustainable materials is contemplated, such as alternative aggregates obtained from recycled concrete, recycled asphalt aggregates, electric arc furnaces, metallurgical slags, and recycled tire materials, such as rubber granules and polypropylene fibers.
- Registering a direct relationship between shrinkage and moisture marks a crucial starting point for applying the results in finite element models. This process involves developing laboratory computational models focused on moisture behavior and drying shrinkage over time. Created from the geometric configuration of the specimens, the boundary conditions and the stress or bending results before and after the failure occurs are required. These factors are vital to developing an advanced pavement model, which integrates constitutive modeling of damaged plasticity in concrete, accompanied by laboratory modeling results, the incorporation of conventional parameters, such as layer stiffness and thickness, interlayer bond, and vehicular loading model, to achieve a more accurate and functional representation of pavement stresses. This last inclusion is crucial for comparison with transfer functions or material behavior models used in the design.

## Figures and Tables

**Table 1:** Roller-compacted concrete mix proportions with 12% cement, 5.65% water, and 0.47 water–cement ratio.

Material	Weight (kg/m^3^)	Volume (m^3^/m^3^)	Volume (L/m^3^)
Cement	288.958	0.092	91.733
Oven-dry weight of aggregates	2119.025	-	-
SSD weight of aggregates	2129	0.767	767.216
Absorbed water	10	-	-
Water at OMC	136.051	-	-
Free water at OMC	126	0.126	126.051
1.5% air	-	0.015	15
Total	2544.034	1	1000

SSD: saturated surface-dry, OMC: optimum moisture content.

**Table 2:** Roller-compacted concrete mix proportions with 16% cement, 5.65% water, and 0.35 water–cement ratio.

Material	Weight (kg/m^3^)	Volume (m^3^/m^3^)	Volume (L/m^3^)
Cement	386.786	0.123	122.789
Oven-dry weight of aggregates	2030.627	-	-
SSD weight of aggregates	2040	0.735	735.210
Absorbed water	10	-	-
Water at OMC	136.584	-	-
Free water at OMC	127	0.127	127.001
1.5% air	-	0.015	15
Total	2553.996	1	1000

SSD: saturated surface-dry, OMC: optimum moisture content.

**Table 3:** Roller-compacted concrete mix proportions with 12% cement, 5.00% water, and 0.42 water–cement ratio.

Material	Weight (kg/m^3^)	Volume (m^3^/m^3^)	Volume (L/m^3^)
Cement	293.247	0.093	93.094
Oven-dry weight of aggregates	2150.5	-	-
SSD weight of aggregates	2161	0.779	778.604
Absorbed water	10	-	-
Water at OMC	122	-	-
Free water at OMC	112	0.112	112.038
Admixture	1.466	0.001264	1.264
1.5% air	-	0.015	15
Total	2567.377	1	1000

SSD: saturated surface-dry, OMC: optimum moisture content.

## Data Availability

Data are contained within the article.

## Abbreviations

The following abbreviations are used in this manuscript:

CEM II/A-M	Portland Composite Cement
f’c	Compressive Strength
GGBF	Ground Granulated Blast Furnace
Mdd	Maximum Dry Density
MR	Modulus of Rupture
OMC	Optimum Moisture Content
RCC	Roller-Compacted Concrete
RCCP	Roller-Compacted Concrete for Pavements
RH	Relative Humidity
SSD	Saturated Surface-Dry
T	Temperature
W/C	Water–Cement Ratio
